# Ultrasensitive and Specific Identification of Monkeypox Virus Congo Basin and West African Strains Using a CRISPR/Cas12b-Based Platform

**DOI:** 10.1128/spectrum.04035-22

**Published:** 2023-02-22

**Authors:** Xu Chen, Wei Yuan, Xinggui Yang, Yuanfang Shi, Xiaoyan Zeng, Junfei Huang, Yi Wang, Shijun Li

**Affiliations:** a The Second Clinical College, Guizhou University of Traditional Chinese Medicine, Guiyang, Guizhou, People’s Republic of China; b Clinical Medical Laboratory of the Second Affiliated Hospital, Guizhou University of Traditional Chinese Medicine, Guiyang, Guizhou, People’s Republic of China; c Department of Quality Control, Guizhou Provincial Center for Clinical Laboratory, Guiyang, Guizhou, People’s Republic of China; d Guizhou Provincial Centre for Disease Control and Prevention, Guiyang, Guizhou, People’s Republic of China; e Experimental Research Center, Capital Institute of Pediatrics, Beijing, People’s Republic of China; City University of Hong Kong

**Keywords:** CRISPR/Cas12b, monkeypox, gold nanoparticle-based lateral flow biosensor, loop-mediated isothermal amplification, point-of-care testing

## Abstract

Human monkeypox (MPX) is a severe and reemerging infectious disease caused by monkeypox virus (MPXV) and forms two distinct lineages, including Congo Basin and West African clades. Due to the absence of specific vaccines and antiviral drugs, developing a point-of-care (POC) testing system to identify MPXV is critical for preventing and controlling MPX transmission. Here, a CRISPR/Cas12b diagnostic platform was integrated with loop-mediated isothermal amplification (LAMP) to devise a novel CRISPR-MPXV approach for ultrasensitive, highly specific, rapid, and simple detection of MPXV Congo Basin and West African strains, and the detection results were interpreted with real-time fluorescence and a gold nanoparticle-based lateral flow biosensor (AuNP-LFB). The optimal detection process, including genomic DNA extraction (15 min), LAMP preamplification (35 min at 66°C), CRISPR/Cas12b-based detection (5 min at 45°C), and AuNP-LFB readout (~2 min), can be completed within 60 min without expensive instruments. Our assay has a limit of detection of 10 copies per test and produces no cross-reaction with any other types of pathogens. Hence, our CRISPR-MPXV assay exhibited considerable potential for POC testing for identifying and distinguishing MPXV Congo Basin and West African strains, especially in regions with resource shortages.

**IMPORTANCE** Monkeypox (MPX), a reemerging zoonotic disease caused by monkeypox virus (MPXV), causes a smallpox-like disease in humans. Early diagnosis is critical to prevent MPX epidemics. Here, CRISPR/Cas12b was integrated with LAMP amplification to devise a novel CRISPR-MPXV approach to achieve highly specific, ultrasensitive, rapid, and visual identification of MPXV Congo Basin and West African strains.

## INTRODUCTION

Monkeypox (MPX) is a severe viral zoonotic disease caused by MPX virus (MPXV), which initially occurred in remote areas of Central and Western Africa (1–3). However, since MPX cases were reported in Europe in early May 2022, an increasing number of cases have been reported from non-African countries (4). More seriously, many of the newly confirmed cases involved no history of travel to regions of endemicity, indicating that community transmission of MPX has begun (5). On 23 July 2022, the World Health Organization (WHO) declared the outbreak of MPX a Public Health Emergency of International Concern (6). MPXV is typically transmitted to humans from infected animals through contact with viral lesions, body fluids, bites, or scratches; human-to-human transmission occurs through respiratory droplets or contact with infected materials (7, 8). The symptoms of MPX in humans are similar to those of smallpox but are milder, and the symptoms include high fever, headache, myalgia, lymphadenopathy, systemic blisters, and rash (9, 10).

MPXV is an enveloped double-stranded DNA virus with ~200 kbp. These base pairs encode approximately 190 proteins to establish viral particles and regulates numerous host processes. In addition, MPXV has been classified as an orthopoxvirus. The virus falls into two distinct clades, the Congo Basin (Central African) clade and West African clade, which show approximately 0.5% genomic sequence difference and have been historically identified in different geographical regions of Africa (9–11). Congo Basin MPX disease has clearly been confirmed to result in a high fatality rate (approximately 10%) and is an important threat to public health (12). Compared to the Congo Basin clade, infections with the West African clade cause less-severe disease in humans, with a case fatality rate of 3.6% (12). The genomic differences between Congo Basin and West African viruses occur in regions that encode important virulence genes and probably explain the differences in clinical severity (11). Although several observational studies have shown that smallpox vaccination provides nearly 85% effectiveness in preventing MPX infection, no specific vaccines or antiviral drugs are available that defend against MPX disease (8). Hence, developing a rapid, sensitive, specific, cost-saving, and easy-to-interpret point-of-care (POC) testing system for the identification and discrimination of MPXV in the Congo Basin and West African strains is important for the prevention and control of MPX transmission.

In recent years, the prokaryotic CRISPR/Cas (clustered regularly interspaced short palindromic repeat and CRISPR-associated protein) system has provided a powerful and promising tool for the development of next-generation nucleic acid diagnostics (13, 14). The principle of CRISPR/Cas testing platforms is based on the *trans*-cleavage activities of Cas effectors, such as Cas12a, Cas12b, Cas13a, and Cas14, which can indiscriminately cleave surrounding single-stranded RNA (ssRNA) and ssDNA when Cas nucleases bind to the target sequence under the guidance of guide RNA (gRNA) (15–18). Compared to traditional nucleic acid amplification tests, CRISPR/Cas-based diagnostic platforms exhibit ultrasensitivity, are highly specific, and do not require specialized instruments (19). To date, several CRISPR/Cas-based detection platforms have been devised and verified, such as CRISPR/Cas12a-assisted nucleic acid detection DETECTR (DNA Endonuclease Targeted CRISPR *trans*-reporter) (20), CRISPR/Cas13a-based molecular detection platform SHERLOCK (Specific High Sensitivity Enzymatic Reporter UnLOCKing) (21), and CRISPR/Cas12b-assisted nucleic acid detection platform HOLMESv2 (One-Hour Low-cost Multipurpose Highly Efficient System v2) (22).

A gold nanoparticle-based lateral flow biosensor (AuNP-LFB), a paper-based diagnostic platform, is widely deemed an ideal tool for POC testing because the sensor is easy to operate, exhibits high reliability and accuracy, produces a rapid response, involves minimal maintenance, shows long-lasting stability, and is economical (23–25). Based on these properties, the AuNP-LFB has been applied to infectious diseases, cancer biomarkers, food safety, and other biomedical fields (26).

In the present study, CRISPRR/Cas12b AuNP-LFB was integrated with loop-mediated isothermal amplification (LAMP) to develop a novel MPXV diagnostic system called CRISPR-MPXV. This system was developed to achieve highly specific, ultrasensitive, rapid, and visual identification of MPXV Congo Basin and West African strains by targeting *D14L* and A-type inclusion body (*ATI*) genes, respectively (27).

## RESULTS

### Overview of the CRISPR-MPXV assay system.

**FIG 1:**
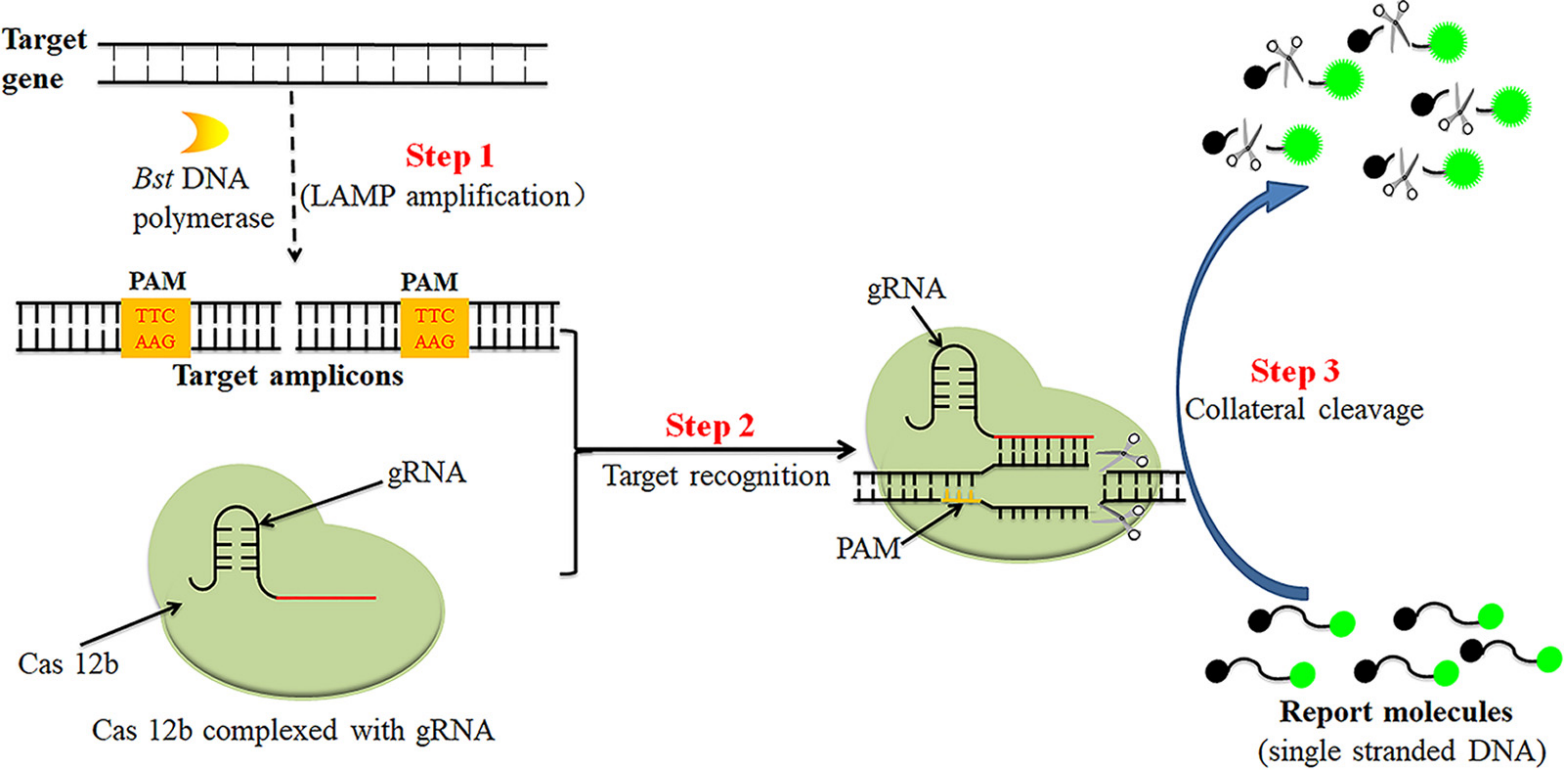
Schematic illustration of the LAMP-CRISPR/Cas12b assay for MPXV detection. The target gene containing a PMA site (TTC) is specifically amplified by the LAMP reaction (step 1). The target amplicons were captured with the gRNA-CRISPR/Cas12b complex through specific gRNA (step 2). Upon recognition of the matching target sequence, the gRNA-CRISPR/Cas12b complex was induced to nonspecifically cleave single-strand DNA reporter molecules (step 3).

**FIG 2:**
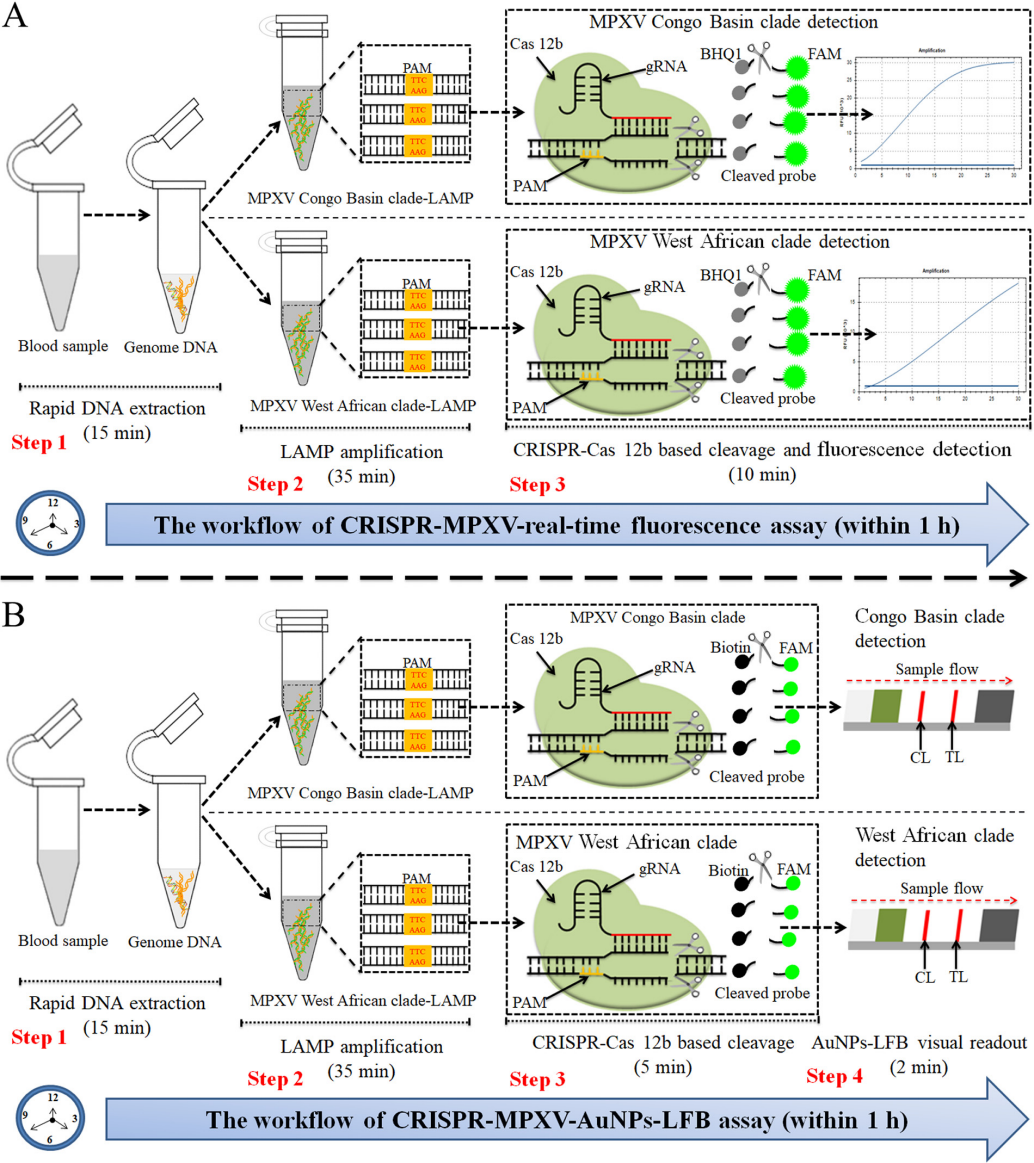
Outline of the CRISPR-MPXV assay workflow. (A) CRISPR-MPXV real-time fluorescence assay. The entire workflow employs the following closely linked steps: rapid DNA extraction (step 1), LAMP amplification (step 2), CRISPR/Cas12b cleavage, and real-time fluorescence readout (step 3). (B) CRISPR-MPXV-AuNP-LFB assay. The entire workflow employs four closely linked steps: rapid DNA extraction (step 1), LAMP amplification (step 2), CRISPR/Cas12b cleavage (step 3), and AuNP-LFB interpretation (step 4).

The principles and workflows of the CRISPR-MPXV assays are presented in Fig. 1 and 2. Briefly, genomic nucleic acid templates were extracted by using viral qEx-DNA/RNA extraction kits (Fig. 2, step 1) and then amplified with LAMP. The MPXV-LAMP amplicons contained the Cas12b PAM site for CRISPR/Cas12b-based identification (Fig. 2, step 2). CRISPR/Cas12b bound to the target sequence under the guidance of the gRNA. The CRISPR/Cas12b effector was then activated for *trans*-cleavage activity, and the ssDNA probes were ultrafast digested (Fig. 2, step 3). The results were monitored simultaneously using real-time fluorescence with Flu-probe 5′-FAM-TTTTTT-BHQ1-3′ and AuNP-LFB with probe 5′-FAM-TTTTTT-Biotin-3′ (Fig. 2).

**FIG 3:**
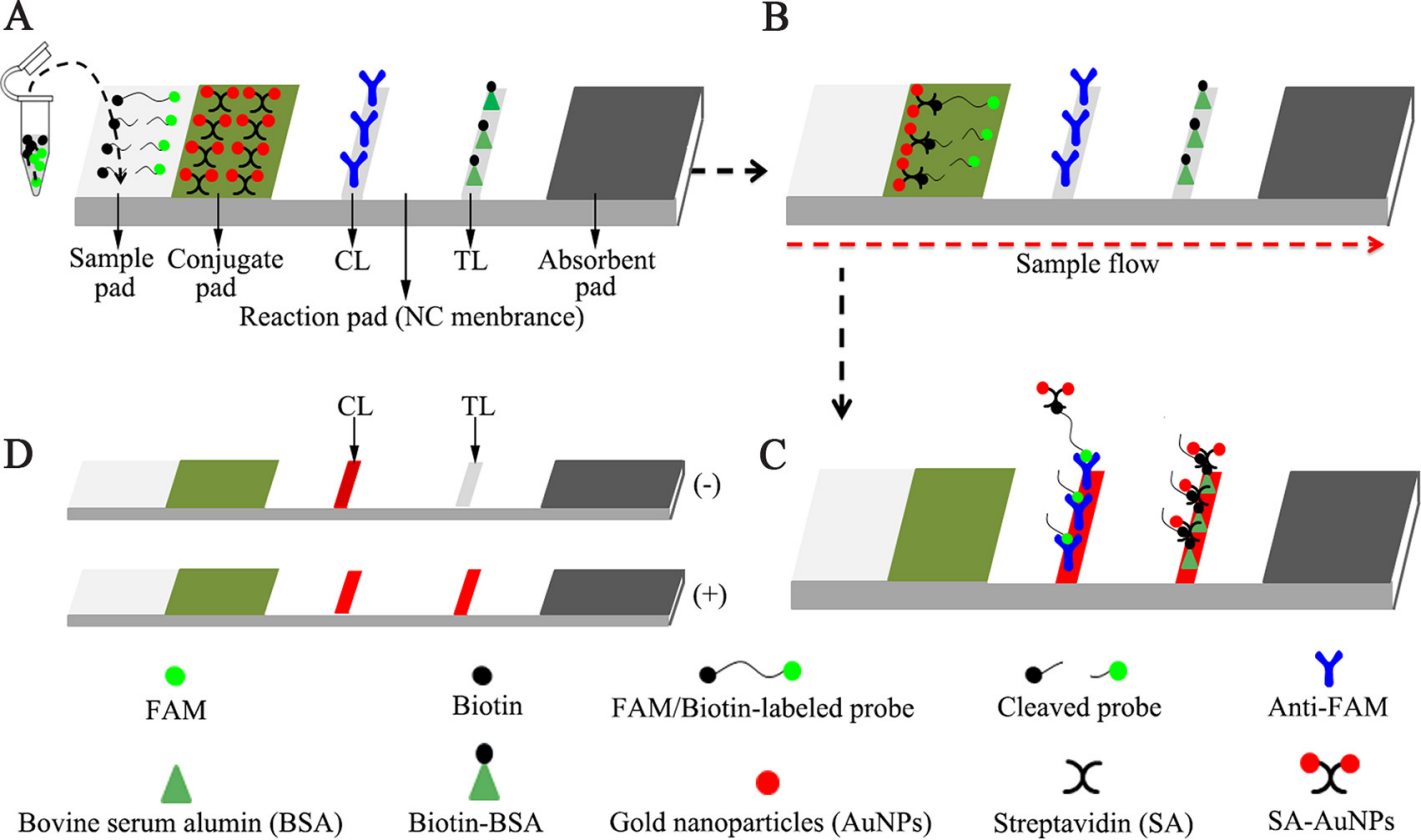
Schematic diagram showing AuNP-LFB principles for the visual interpretation of CRISPR-MPXV outcomes. (A) CRISPR-MPXV reaction products (2.0 μL) and running buffer (100 μL) were simultaneously deposited on the sample pad. (B) Due to capillary action, the running buffer containing CRISPR-MPXV products moved forward onto the conjugate pad and nitrocellulose (NC) membrane. Meanwhile, the dye streptavidin-coated gold nanoparticles (SA-AuNP) were rehydrated in the conjugate region. Then, the biotin-labeled probes were combined with SA-AuNP at the conjugate pad. (C) For positive results, the ssDNA probes (5′-FAM-TTTTTT-Biotin-3′) were *trans*-cleaved by activated-CRISPR/Cas12b nuclease, and the FAM and biotin were separated. Hence, the biotin-SA-AuNP complexes were captured by biotin-BSA at the test line (TL). In the negative outcome, the ssDNA probes were not cleaved and were specifically arrested by anti-FAM at the control line (CL). Therefore, the biotin of ssDNA probes combined SA-AuNP for visualization at CL. (D) Interpretation of the CRISPR-MPXV assay results. For positive outcomes, CL and TL appeared simultaneously on the AuNP-LFB. For negative results, only the CL line was observed on the AuNP-LFB.

For AuNP-LFB identification, CRISPR-MPXV products (2.0 μL) and 100 μL of running buffer (100 mM phosphate-buffered saline [PBS; pH 7.4]) were concurrently added to an AuNP-LFB sample pad (Fig. 3A). The CRISPR-MPXV product-containing flowing buffer moved along the AuNP-LFB through capillary action, and the crimson red dye streptavidin-AuNP was rehydrated on the conjugate pad (Fig. 3B). For a positive result, the ssDNA probe molecules were *trans*-cleaved with activated CRISPR/Cas12b nuclease, and FAM and biotin were separated. The biotin-streptavidin-AuNP complexes were then seized by using biotinylated bovine serum albumin (biotin-BSA) at the test line (TL) (Fig. 3C). For a negative outcome, the ssDNA probe molecules were not cleaved, and the crimson red dye streptavidin-AuNP-ssDNA probes were specifically captured by anti-FAM at the control line (CL) (Fig. 3C). The interpretation of the CRISPR-MPXV assay using AuNP-LFB is outlined in Fig. 3D, as well as Fig. S2 in the supplemental material.

### Optimal reaction conditions for the CRISPR-MPXV assay.

The incubation temperature is critical for LAMP amplification, and the reaction temperature for the LAMP preamplification stage was optimized to range from 61 to 69°C with *D14L* and *ATI* plasmids (1.0 × 10^4^ copies). The outcomes of the LAMP reaction were tracked with real-time turbidity using a real-time turbidity monitoring device (LA-500). The results indicated that the robust amplification of *D14L*-LAMP and *ATI*-LAMP occurred at 66°C (see Fig. S3A and B).

Multiple assay reaction times (1, 2, 5, 10, and 20 min) were tested to confirm the optimal reaction time for CRISPR/Cas12b detection. Real-time fluorescence and AuNP-LFB were simultaneously used to track the outcomes. The results showed that a stable visual signal of AuNP-LFB and fluorescent signal were detected within 5 min (see Fig. S4A to D).

### Sensitivity and specificity of the CRISPR-MPXV assay.

**FIG 4:**
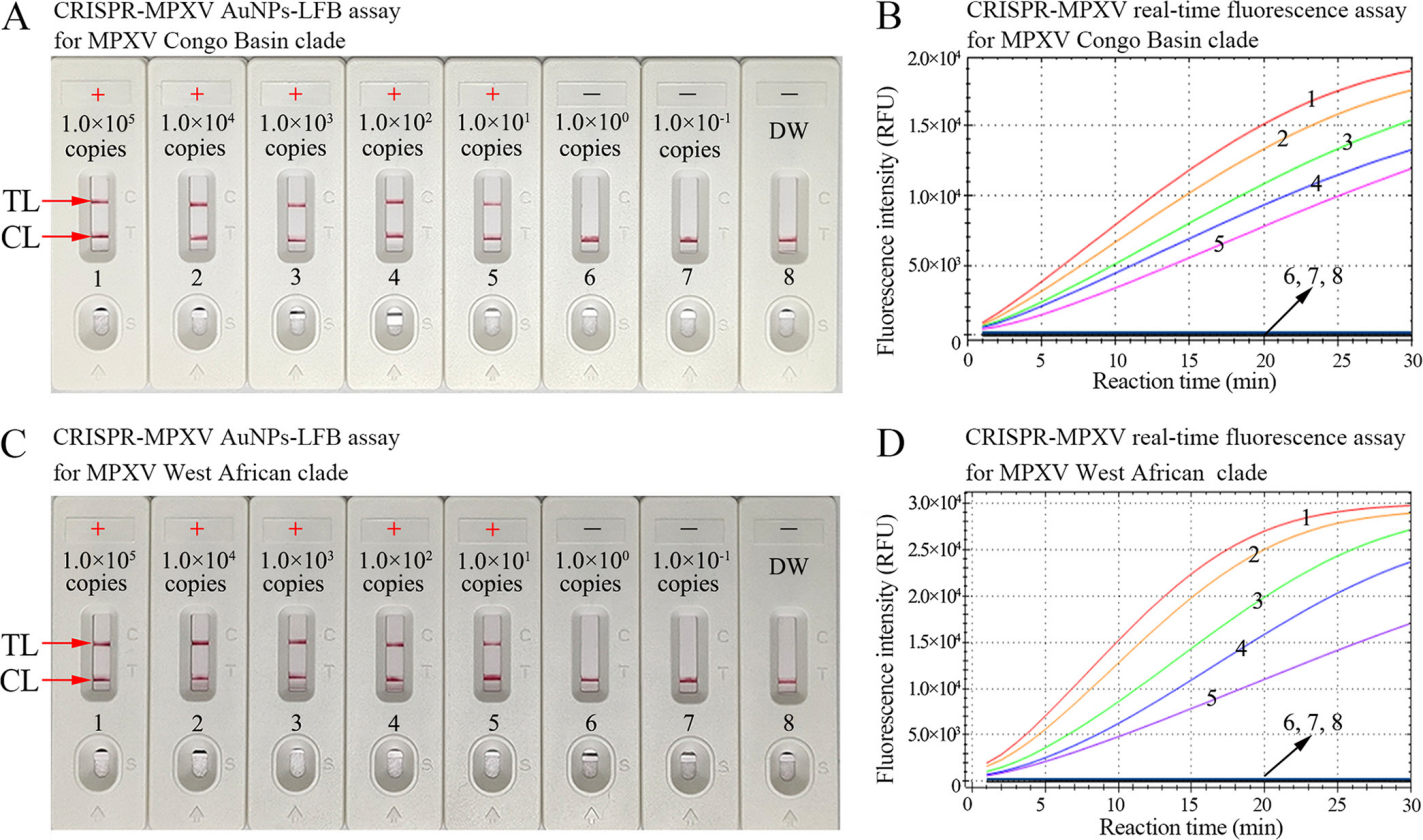
Sensitivity of the CRISPR-MPXV assay. AuNP-LFB and real-time fluorescence (RTF) approaches were simultaneously used to readout the CRISPR-MPXV outcomes. (A and B) AuNP-LFB (A) and RTF (B) biosensor samples 1 to 8 represent the MPXV Congo Basin clade *D14L* plasmid concentrations of 1 × 10^5^, 1 × 10^4^, 1 × 10^3^, 1 × 10^2^, 1 × 10^1^, 1 × 10^0^, and 1 × 10^−1^ copies per reaction and negative control (distilled water [DW]), respectively. (C and D) AuNP-LFB (C) and RTF (D) biosensor samples 1 to 8 represent MPXV West African clade *ATI* plasmid concentrations of 1 × 10^5^, 1 × 10^4^, 1 × 10^3^, 1 × 10^2^, 1 × 10^1^, 1 × 10^0^, and 1 × 10^−1^ copies per reaction and negative control (DW), respectively. The limit of detection of the CRISPR-MPXV assay was 10 copies per reaction. +, positive; −, negative; CL, control line; TL, test line.

Serial dilutions of nucleic acid templates (*D14L* and *ATI* plasmids) ranging from 5.0 × 10^4^ to 5.0 × 10^−1^ copies were used to identify the limitation of detection of our assay. CRISPR-MPXV assays were carried out as described above, and the outcomes were monitored by real-time fluorescence and AuNP-LFB. As shown in Fig. 4, the sensitivity of the assay was 10 copies per test for both the *D14L* plasmid and *ATI* plasmid, and the visual AuNP-LFB readouts were consistent with the real-time fluorescence detections.

**FIG 5:**
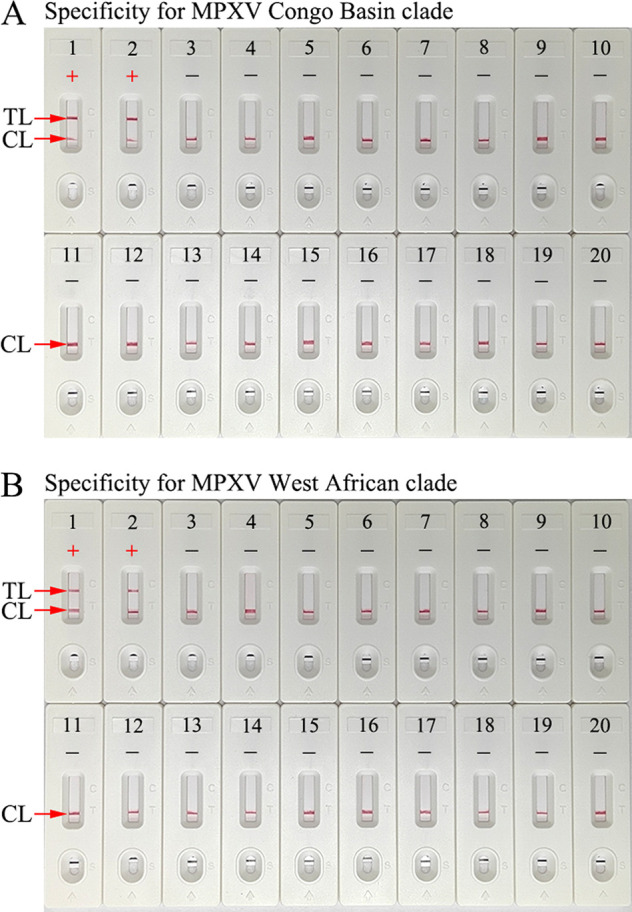
Specificity of the CRISPR-MPXV assay. (A) Specificity of the CRISPR-MPXV-AuNP-LFB assay for MPXV Congo Basin clade detection. Biosensor 1, MPXV Congo Basin-*D14L*-plasmid; biosensor 2, MPXV Congo Basin pseudovirus; biosensor 3, herpes simplex virus type 2 (HSV-2); biosensor 4, cytomegalovirus; biosensor 5, Epstein-Barr virus; biosensor 6, measles virus; biosensor 7, Sendai virus; biosensor 8, hepatitis B virus; biosensor 9, parvovirus; biosensor 10, parainfluenza virus type 3; biosensor 11, human enterovirus EV71; biosensor 12, coxsackievirus CAV16; biosensor 13, *Adenoviridae*; biosensor 14, influenza B virus; biosensor 15, influenza A virus; biosensor 16, herpes zoster virus; biosensor 17, human papillomavirus; biosensor 18, M. tuberculosis; biosensor 19, *Neisseria gonorrhoeae*; biosensor 20, negative control (distilled water, DW). CL, control line; TL, test line; +, positive; −, negative. (B) Specificity of the CRISPR-MPXV-AuNP-LFB assay for MPXV West African clade detection. biosensor 1, MPXV West African-*ATI*-plasmid; biosensor 2, MPXV West African pseudovirus; biosensor 3, herpes simplex virus type 2 (HSV-2); biosensor 4, cytomegalovirus; biosensor 5, Epstein-Barr virus; biosensor 6, measles virus; biosensor 7, Sendai virus; biosensor 8, hepatitis B virus; biosensor 9, parvovirus; biosensor 10, parainfluenza virus type 3; biosensor 11, human enterovirus EV71; biosensor 12, coxsackievirus CAV16; biosensor 13, *Adenoviridae*; biosensor 14, influenza B virus; biosensor 15, influenza A virus; biosensor 16, herpes zoster virus; biosensor 17, human papillomavirus; biosensor 18, M. tuberculosis; biosensor 19, *N. gonorrhoeae*; biosensor 20, negative control (distilled water [DW]). CL, control line; TL, test line; +, positive; −, negative.

The specificity of the CRISPR-MPXV assay was verified by testing *D14L* plasmid, *ATI* plasmid, MPXV-*D14L* pseudovirus, MPXV-*ATI* pseudovirus, and 17 other microbes (Table 1). The CRISPR-MPXV assay protocol was performed as described above, and the results were read with real-time fluorescence and AuNP-LFB. The positive outcomes appeared only for the templates extracted from the MPXV Congo Basin or West African clade, while non-MPXV microbes and the blank control presented negative outcomes (Fig. 5; see also Fig. S5 and S6), and no cross-reactions were observed from the CRISPR-MPXV assay. Therefore, our assay accurately detected and distinguish MPXV Congo Basin and West African clades.

**TABLE 1:** Strains and synthetic templates used in the present study

Strain or template	Source^*a*^	No. of strains	CRISPR-MPXV assay^*b*^			
			AuNP-LFB		RFA	
			CB	WA	CB	WA
MPXV Congo Basin clade *D14L* plasmids	Synthesized by Tsingke Biotech (Beijing, China)	1	P	N	P	N
MPXV Congo Basin clade (pseudovirus)	Synthesized Sangon Biotech (Shanghai, China)	1	P	N	P	N
MPXV West African clade *ATI* plasmids	Synthesized by Tsingke Biotech (Beijing, China)	1	N	P	N	P
MPXV West African clade (pseudovirus)	Synthesized Sangon Biotech (Shanghai, China)	1	N	P	N	P
Herpes simplex virus	CHCIP	1	N	N	N	N
Cytomegalovirus	CHCIP	1	N	N	N	N
Epstein-Barr virus	2nd GZUTCM	1	N	N	N	N
Measles virus	CHCIP	1	N	N	N	N
Sendai virus	CHCIP	1	N	N	N	N
Hepatitis B virus	2nd GZUTCM	1	N	N	N	N
Parvovirus	CHCIP	1	N	N	N	N
Parainfluenza virus	CHCIP	1	N	N	N	N
Human enterovirus EV71	GZCDC	1	N	N	N	N
Coxsackievirus CAV16	GZCDC	1	N	N	N	N
Adenoviridae	CHCIP	1	N	N	N	N
Influenza B virus	CHCIP	1	N	N	N	N
Influenza A virus	CHCIP	1	N	N	N	N
Herpes zoster virus	CHCIP	1	N	N	N	N
Human papilloma virus	2nd GZUTCM	1	N	N	N	N
M. tuberculosis	GZCDC	1	N	N	N	N
N. gonorrhoeae	2nd GZUTCM	1	N	N	N	N

a CHCIP, Children’s Hospital Capital Institute of Pediatrics; 2nd GZUTCM, the Second Affiliated Hospital, Guizhou University of Traditional Chinese Medicine; GZCDC, Guizhou Provincial Center for Disease Control and Prevention.

b RFA, real-time fluorescence; P, positive; N, negative. The results are expressed per Congo Basin (CB) or West African (WA) clade.

### Evaluating the feasibility of the CRISPR-MPXV assay using simulated clinical samples.

Next, the feasibility of the CRISPR-MPXV assay to accurately identify and discriminate MPXV Congo Basin and MPXV West African strains was confirmed. Sixty simulated clinical samples (30 skin swabs and 30 throat swabs) were simultaneously detected using DNA sequencing and our assay. The DNA sequencing results showed that 12 skin swabs and 9 throat swabs were verified as belonging to the MPXV Congo Basin clade, and 8 skin swabs and 16 throat swabs were verified as belonging to the MPXV West African clade. These results were consistent with our CRISPR-MPXV assay outcomes (Table 2). Thus, these data indicated that the CRISPR-MPXV assay functioned as an advanced diagnostic tool to identify and discriminate MPXV Congo Basin and West African strains.

**TABLE 2:** Comparison of CRISPR-MPXV and DNA sequencing methods to identify MPXV in simulated clinical samples

Detection method	No. of swabs^*a*^					
	Simulated skin swabs (*n* = 30)			Simulated throat swabs (*n* = 30)		
	CB	WA	Negative	CB	WA	Negative
DNA sequencing	12	8	10	9	16	5
CRISPR-MPXV-AuNP-LFB	12	8	10	9	16	5
CRISPR-MPXV-RTF	12	8	10	9	16	5

a The results are expressed, where applicable, per Congo Basin (CB) or West African (WA) clade.

## DISCUSSION

In our study, a novel CRISPR-MPXV diagnosis platform that combined CRISPRR/Cas12b-based testing with LAMP preamplification was successfully devised for the ultrasensitive, highly specific, rapid and visual identification and discrimination of MPXV Congo Basin and West African strains. The feasibility of our platform was verified using simulated clinical samples, and the results were compared to DNA sequencing. Multiple laboratory diagnostic approaches have been reported to detect monkeypox virus, such as cultivation, electron microscopy, and nucleic acid amplification technologies (NAATs) (27–29). Culture and electron microscopy were not recommended since high containment facilities and specific facilities were needed. With respect to high specificity and sensitivity, real-time PCR is currently the gold standard for diagnosing monkeypox virus (29). Li et al. used real-time PCR to specifically identify West African and Congo Basin monkeypox virus strains and detected 3.5 and 40.4 copies, respectively (29). However, high-precision thermal cyclers and well-established laboratory infrastructures are needed, which limits accessibility in resource-poor settings. Here, our CRISPR-MPXV diagnostic system can perform isothermally without specialized instruments, and then the results can be visually readout using AuNP-LFB. The CRISPR-MPXV detection procedure, including nucleic acid extraction (15 min), LAMP preamplification (35 min), CRISPR/Cas12b detection (5 min), and AuNP-LFB visual result interpretation (within 2 min), can be completed within 60 min.

CRISPR/Cas systems were initially discovered in the adaptive immunity of bacteria and archaea for defense against invasion nucleic acids (30, 31). Over the last few years, many CRISPR/Cas systems, such as CRISPR/Cas12, CRISPR/Cas9, and CRISPR/Cas13, have been widely applied in biomedical fields for gene editing (30, 32). The RNA-guided cleavage of Cas effectors is flexibly utilized through a simple redesign of spacer sequences for different target genes (32). Recently, CRISPR/Cas systems have been repurposed and show promise for the development of robust diagnostic tools to detect nucleic acids, these systems exhibit unique characteristic, including collateral cleavage for target genes and nonspecific single-stranded nucleic acids (13, 17). The ultrasensitivity, precision, and specificity of CRISPR/Cas-based detection could be due to target-dependent gRNA. In the present study, specific gRNAs for MPXV West African and Congo Basin strains were successfully devised, and the gRNAs efficiently navigated Cas12b effectors to each of the target sequences. The specificity of the CRISPR-MPXV assay was verified with MPXV strains and other pathogens. Owing to the lack of reference strains for the MPXV West African and Congo Basin clades in our laboratory, the full-length Congo Basin clade *D14L* gene and the West African clade *ATI* gene were extracted from synthetically produced MPXV-*D14L* pseudovirus and MPXV-*ATI* pseudovirus, respectively. Each of them acted as a positive control. As expected, the CRISPR-MPXV detection system clearly distinguished MPXV West African and Congo Basin strains and showed no cross-reaction with other pathogens (Fig. 5; see also Fig. S5 and S6). Hence, our CRISPR-MPXV assay was extremely specific for identifying and distinguishing MPXV West African and Congo Basin strains. To test the sensitivity of the CRISPR-MPXV assay, each of the synthesized plasmids was diluted from 1.0 × 10^5^ to 1.0 × 10^−1^ copies/test. The results confirmed that our novel CRISPR-MPXV assay can detect as few as 10 copies of genomic DNA per test. More importantly, we also successfully applied the CRISPR-MPXV assay to test simulated clinical samples. The artificial samples were simultaneously detected with CRISPR-MPXV and DNA sequencing methods, and concordance results were obtained between the former and latter assays. A shortcoming of our study is that monkeypox-positive clinical samples were not collected to further confirm the feasibility of our assay, as monkeypox cases are lacking in China to date. Fortunately, pseudoviruses can verify the feasibility of nucleic acid detection technologies (33, 34).

In the present study, loop-mediated isothermal amplification (LAMP) was applied to preamplify the target sequences (MPXV Congo Basin clade *D14L* gene and West African clade *ATI* gene). LAMP is a novel nucleic acid isothermal amplification approach that can robustly amplify the target sequence at a constant temperature (58 to 69°C) for 30 to 60 min using Bacillus stearothermophilus DNA polymerase with four or six primer sets that span six or eight unique sections of the target gene (35–37). The primer set contained forward and reverse outer (F3 and B3) and inner (FIP and BIP) primers, and the four primers were usually sufficient for LAMP amplification. The two extra loop primers (LF and LB) are usually incorporated into the LAMP reaction to improve the reaction’s efficiency and specificity. Here, two sets of LAMP primers were successfully designed to target the *D14L* and *ATI* genes (Table 3; see also Fig. S1). The optimal LAMP reaction conditions were determined to be 66°C for 35 min (see Fig. S3).

**TABLE 3:** Primers and gRNAs used in the present study

Clade and primer/gRNA^*a*^	Sequence (5′–3′)	Length^*b*^
MPXV Congo Basin clade		
F3	GGAATCCTGAGGCACCTA	18 nt
B3	ACATTTAACAATCTGACACGT	21 nt
FIP	CTCCCATCGATATAAAAATCCTCGTATCTGTTAAATGCCAATCACC	46 mer
BIP	AGTTGCAATAGTGGATATTCGTTGAGACCATTCTCCTCCTGAAC	44 mer
LF	CCGTTATGTCTTCCGTTG	18 nt
LB	TGGTAACTCTGGTGTCAT	18 nt
gRNA	GUCUAGAGGACAGAAUUUUUCAACGGGUGUGCCAAUGGCCACUUUCCAGGUGGCAAAGCCCGUUGAGCUUCUCAAAUCUGAGAAGUGGCACGUUGAUUGGUAACUCUGGUG	111 mer
MPXV West African clade		
F3	AGGAGGTAAATAGGCTAAAGAC	22 nt
B3	GCGAGTCAATTCCCTCCTA	19 nt
FIP	CCGAATAGAGTTCTGATTCATCCTTTAGAATCAGGGATCTTGAACG	46 mer
BIP	TAAAACTGAACTCGGTAATGCCAGTTGTCAGATTCACGCTCTC	43 mer
LF	GAGAAGATCTCTAGCGA	17 nt
LB	AGTAACTTGCAAGAAAGTC	19 nt
gRNA	GUCUAGAGGACAGAAUUUUUCAACGGGUGUGCCAAUGGCCACUUUCCAGGUGGCAAAGCCCGUUGAGCUUCUCAAAUCUGAGAAGUGGCACCCUGGCAUUACCGAGUUCAG	111 mer

a The target genes, GenBank accession numbers, and nucleotide positions for each clade were as follows: (i) MPXV Congo Basin clade—*D14L*, KP849471.1, (19333 to 19544) and (ii) MPXV West African clade—*ATI*, MT903346.1, 135637 to 135839.

b nt, nucleotide; mer, monomeric unit.

For a visual readout of CRISPR-MPXV results, AuNP-LFB was used in our diagnosis system. AuNP-LFB is a paper-based detection platform and is promising for POC testing because it is easy to manufacture, exhibits specificity and sensitivity, is inexpensive and simple to operate, and provides a visual readout (38, 39). The AuNP-LFB can visually interpret the CRISPR-MPXV outcomes for labeling with anti-FAM and BSA-biotin on the nitrocellulose membrane. For positive outcomes, the ssDNA probes (5′-FAM-TTTTTT-Biotin-3′) were *trans*-cleaved by the activated CRISPR/Cas12b nuclease, the FAM and biotin probes were separated, and the biotin-streptavidin-AuNP complexes were captured by biotin-BSA and visually read at the test line (TL). For negative outcomes, the ssDNA probes were not cleaved, and the FAM/biotin-labeled probe-streptavidin-AuNP complexes were arrested by anti-FAM at the control line (CL) (Fig. 3). In the present study, a real-time fluorescence technique was also applied to interpret the CRISPR-MPXV results. However, interpreting CRISPR-MPXV results with real-time fluorescence techniques requires expensive devices, which may not be available in many resource-limited regions.

In conclusion, CRISPRR/Cas12b was successfully integrated with LAMP amplification to devise a novel CRISPR-MPXV approach for highly specific, ultrasensitive, rapid, and visual identification and discrimination of MPXV Congo Basin and West African strains. Our assay exhibited a 10-copy limit of identification and showed no cross-reaction with any other pathogens. The entire testing process can be completed within 60 min and does not necessitate any sophisticated devices. Hence, these characteristics matched the WHO-recommended POC testing requirements (low cost, sensitive, specific, user-friendly, robust, equipment-free, and attainable).

## MATERIALS AND METHODS

### Reagents.

AacCRISPR/Cas12b protein (C2c1) and universal isothermal amplification kits were obtained from HuiDeXin Biotech Co., Ltd. (Tianjin, China). Gold nanoparticle-based lateral flow biosensor (AuNP-LFB) materials, including crimson red dye streptavidin-coated AuNP (SA-AuNP, 40 ± 5 nm), were purchased from Bangs Laboratories, Inc. (Fishers, IN), rabbit anti-fluorescein antibody (anti-FAM), and biotinylated bovine serum albumin (biotin-BSA) were obtained from Abcam Co., Ltd. (Shanghai, China). Four AuNP-LFB sections, including the sample pad, conjugation pad, nitrocellulose membranes, and absorption pad, were manufactured and laminated on plastic adhesive backing by HuiDeXing Biotech Co., Ltd., according to our design scheme (Fig. 3).

### Target DNA and artificial MPXV virus preparation.

Full-length Congo Basin clade *D14L* (accession no. KP849471.1) and West African clade *ATI* (accession no. MT903346.1) sequences were synthetically produced and cloned into the pUC57 vector (Tsingke Biotech, China). The initial concentration of each plasmid was 1 × 10^8^ copies, and the two constructed plasmids acted as positive controls.

Two pseudoviruses (MPXV-*D14L* pseudovirus and MPXV-*ATI* pseudovirus) were constructed by Sangon Biotech Co., Ltd. (Shanghai, China), which were made with 293A cell cultures and included segments of the *D14L* gene (GenBank accession no. KP849471.1, genome coordinates 19294 to 19944) and *ATI* gene (GenBank accession no. MT903346.1, genome coordinates 135527 to 135918), respectively. Genomic DNA was extracted using viral qEx-DNA/RNA extraction kits (Xi′an Tianlong Science & Technology Co., Ltd., Xian, China) in accordance with the manufacturer’s instructions. The concentrations of genomic DNA were measured using a NanoDrop ND-2000 instrument (Thermo, USA) at *A*_260/280_. Other microorganisms used in this study are shown in Table 1.

### LAMP primer and gRNA design.

The MPXV Congo Basin and West African clade LAMP primers were designed using software programs Primer Explorer V5 (http://primerexplorer.jp/e/) and PRIMER PREMIER 5.0 in accordance with the principle of LAMP reaction based on the MPXV Congo Basin-specific *D14L* gene and MPXV West African-specific partial *ATI* gene, respectively (27). The specificity of each LAMP primer was verified with the BLAST analysis tool. Two gRNAs for the MPXV Congo Basin *D14L* gene and West African *ATI* gene were designed according to the CRISPR/Cas12b detection mechanism. The locations of each LAMP primer and gRNA are shown in Fig. S1. The principle of LAMP preamplification and CRISPR/Cas12b-based assay is shown in Fig. 1. The sequences of LAMP primers and gRNAs are shown in Table 3. All the oligonucleotides were synthesized and purified by GenScript Biotech Co., Ltd. (Nanjin, China), with high-performance liquid chromatography purification grade.

### LAMP amplification.

LAMP preamplification was performed in a 25-μL reaction volume. Briefly, 12.5 μL of 2× reaction buffer [2 M betaine, 16 mM MgSO_4_, 40 mM KCl, 20 mM (NH_4_)_2_SO_4_, 40 mM Tris-HCl (pH 8.8), and 0.2% Tween 20]; 1 μL of *B. stearothermophilus* 2.0 DNA polymerase (8 U); 0.8 μM concentrations each of F3, B3, LF, and LB; 1.6 μM concentrations each of FIP and BIP; and 2 μL for each genomic DNA template, adding double-distilled water up to 25 μL. The LAMP reaction was carried out using a heat blocker, and the amplification results were measured with real-time turbidity using a real-time turbidity monitoring device (LA-500) to optimize the reaction temperature.

### CRISPR/Cas12b-based identification.

In this study, AacCas12b (C2c1) was used for CRISPR/Cas-based *trans*-cleavage identification. In brief, the CRISPR/Cas12b-gRNA complexes were preassembled in a 50-μL reaction volume, which included 2 μL of AacCas12b (100 pM), 2 μL of gRNA (10 μM), and 25 μL of 2 × HDX buffer, adding distilled water (DW) up to 50 μL, and then the mixtures were preincubated at 37°C for 10 min. The CRISPR/Cas12b-gRNA complexes were used immediately or stored at low temperatures (0 to 4°C) for no more than 24 h before use.

A CRISPR/Cas12b *trans*-cleavage assay was performed as follows (25-μL reaction volume): 12.5 μL of 2× HDX buffer, 2 μL of LAMP products, 4 μL of CRISPR/Cas12b-gRNA complex, 1 μL of ssDNA reporter molecule, and DW up to 25 μL. The detection process was performed at 45°C for 5 min. The results were analyzed by using real-time fluorescence and AuNP-LFB. Flu-probe (5′-FAM-TTTTTT-BHQ1-3′, 100 μM) was used for real-time fluorescence analysis. For the AuNP-LFB assay, the probe was replaced with 5′-FAM-TTTTTT-Biotin-3′ (50 μM).

### AuNP-LFB preparation and assay.

The gold nanoparticle-based lateral flow biosensor (AuNP-LFB; size, 60 × 4 mm) is illustrated in Fig. 3. In brief, the AuNP-LFB was composed of four sections, including nitrocellulose membrane, sample, conjugate, and absorption pads. Crimson red dye streptavidin-gold nanoparticles (SA-GNPs) were deposited onto the conjugate pad. Anti-FAM (4 mg mL^−1^) and biotin-BSA (0.2 mg mL^−1^) were immobilized onto the nitrocellulose membrane for the control line (CL) and test line (TL), respectively, with 5 mm separating each line. For the AuNP-LFB assay, 2.0 μL of CRISPR/Cas12b *trans*-cleavage products and 100 μL of running buffer (100 mM PBS, 1% Tween 20 [pH 7.4]) were simultaneously added to the sample pad, and the running buffer containing ssDNA reporter molecule solution flowed along the AuNP-LFB via capillary action. Finally, the results were generated on the nitrocellulose membrane (red line) within 2 min.

### Sensitivity and specificity of the CRISPR-MPXV assay.

Two standard plasmids, the *D14L* and *ATI* plasmids, were serially diluted by 10-fold ranging from 1.0 × 10^5^ to 1.0 × 10^−1^ copies. CRISPR-MPXV reactions were performed as previously described, and the results were monitored with real-time fluorescence and AuNP-LFB. Each dilution was tested at least three times.

To verify the specificity of our assay, pseudoviruses of MPXV and other microbes (Table 1) were used for CRISPR-MPXV detection, and all of the results were read simultaneously with real-time fluorescence and AuNP-LFB. All examinations were tested at least three times.

### Verifying the feasibility of the CRISPR-MPXV assay using simulated clinical samples.

Owing to the lack of MPXV clinical samples, two kinds of pseudoviruses, MPXV-*D14L* pseudovirus and MPXV-*ATI* pseudovirus, were used to verify the feasibility of our assay. Briefly, 30 human skin swabs and 30 throat swabs were collected from healthy volunteers, and each skin swab or throat swab was fully immersed in 200 μL of sterile virus preservation medium (DaAn Gene Co., Ltd., China). Then, 10 to 500 copies of each pseudovirus were randomly added to each solution. In compliance with the producer’s guidelines, genomic DNA templates were obtained through viral qEx-DNA/RNA extraction kits (Xi′an Tianlong Science & Technology Co.). The CRISPR-MPXV operation was performed as described above. In addition, all artificial samples were tested using DNA sequencing (Tsingke Biotech). Finally, the results of the CRISPR-MPXV assay were compared to those from DNA sequencing.

### Ethics statement.

This study was approved by the Human Ethics Committee of the Second Affiliated Hospital, Guizhou University of Traditional Chinese Medicine (approval KYW2022009), and complied with the Declaration of Helsinki. Before our team obtained healthy-person samples and conducted this study, all personal patient identifiers were removed. Patient informed consent was waived by the ethics committee.

### Data availability.

The data sets used and/or analyzed during the present study are available from the corresponding author on reasonable request.
