# Supplementary material for: Ultrasensitive and Specific Identification of Monkeypox Virus Congo Basin and West African Strains Using a CRISPR/Cas12b-Based Platform
Source: Microbiol Spectr. 2023 Feb 22;11(2):e04035-22. doi: 10.1128/spectrum.04035-22 (PMC10100855; doi:10.1128/spectrum.04035-22)
Supplement: Supplemental file 1 — Fig. S1 to S6. Download spectrum.04035-22-s0001.pdf, PDF file, 1.5 MB [file spectrum.04035-22-s0001.pdf]

Ultrasensitive and Specific Identification of Monkeypox Virus Congo Basin and West African Strain Using CRISPR/Cas12b-Based Platform

Xu Chen[a, b*], Wei Yuan[c], Xinggui Yang[d], Yuanfang Shi[a], Xiaoyan Zeng[a, b], Junfei Huang[d], Yi Wang[e*], and Shijun Li[d*]

[a]The Second Clinical College, Guizhou University of Traditional Chinese Medicine, Guiyang, Guizhou, 550003, People's Republic of China

[b]Clinical Medical Laboratory of the Second Affiliated Hospital, Guizhou University of Traditional Chinese Medicine, Guiyang, Guizhou, 550003, People's Republic of China

[c]Department of Quality Control, Guizhou Provincial Center for Clinical Laboratory, Guiyang, Guizhou, 550002, People's Republic of China

[d]Guizhou Provincial Centre for Disease Control and Prevention, Guiyang, Guizhou, 550004, People's Republic of China

[e]Experimental Research Center, Capital Institute of Pediatrics, Beijng 100020, People's Republic of China

[*]Corresponding author:

Shijun Li, E-mail: zjumedjun@163.com

Yi Wang, E-mail: wildwolf0101@163.com

Xu Chen, E-mail: xuchen1220@126.com

SUPPLEMENTARY MATERIALS

The Supplementary materials include sequences and location of MPXV Congo Basin clade *DL14* gene and MPXV West African clade *ATI* gene used to design LAMP primers and gRNAs (**Fig. S1**); Interpretation of CRISPR-MPXV gold nanoparticle-based lateral flow biosensor results (**Fig. S2**); Optimization of the temperature for the MPXV Congo Basin clade-and MPXV West African clade-LAMP amplification (**Fig. S3**); Optimization of the reaction time for CRISPR–Cas12b/gRNA cleavage (**Fig. S4**); Specificity of CRISPR-MPXV real-time fluorescence detection (**Fig. S5 and S6**).

Figure legends

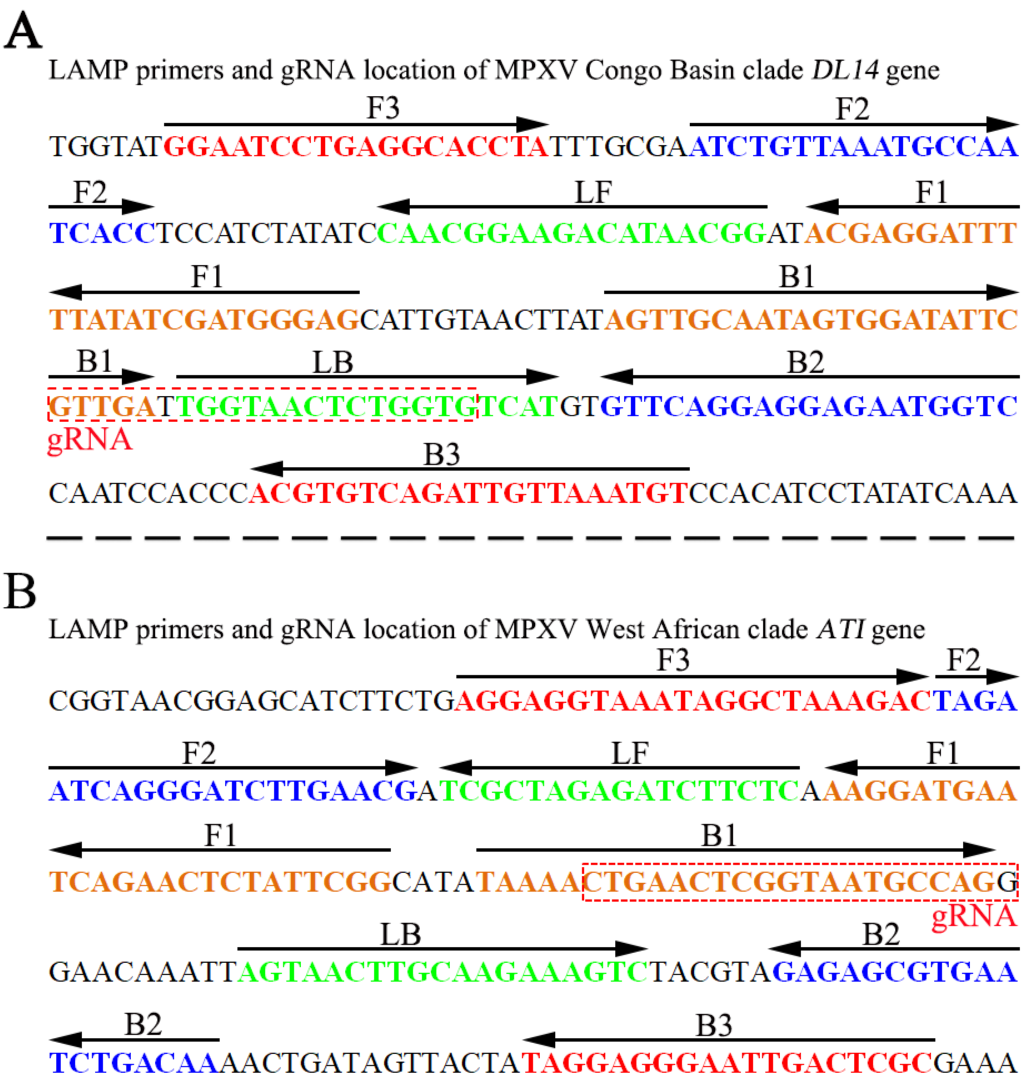

Fig. S1 Sequences and location of MPXV Congo Basin clade *DL14* gene (**A**) and MPXV

West African clade *ATI* gene (**B**) used to design LAMP primers and gRNAs. The sites of LAMP primers were underline, and the gRNAs were in boxed. Right arrows and left arrows indicated the sense and complementary sequences which were used in this study, respectively.

Schematic	Example	Description
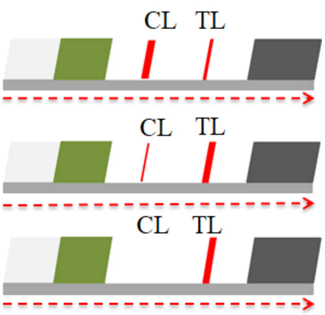	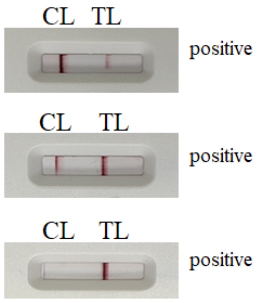	For positive results, there will be an easy to observe crimson band at the test line (TL). In some cases, the TL will be weaker than the control line (CL).<br><br>The CL may not be observed when the ssDNA probers are complete digested by Cas 12b.
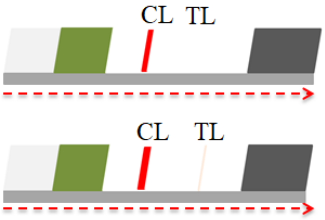	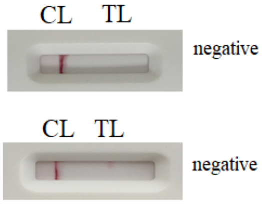	For negative results, there have no signal at the test line (TL), and a crimson band appears on the control line (CL).<br><br>Negative strips may exhibit a faint signal at TL when put the biosensor at room temperature over 10 minutes, but the signal is much fainter than a real positive signal.

Fig. S2 Instructions for the interpretation of CRISPR-MPXV gold nanoparticle-based lateral flow biosensor results.

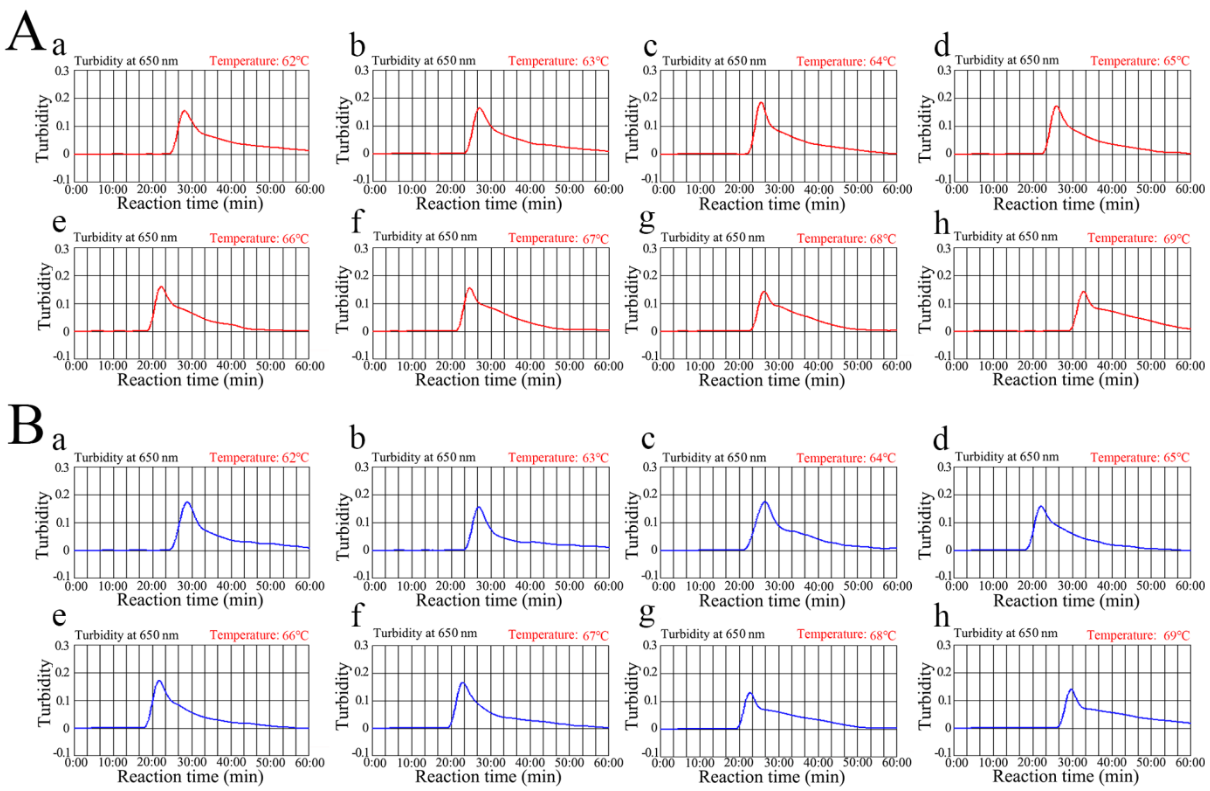

Fig. S3 Optimizing of the temperature for the MPXV Congo Basin clade-and MPXV West African clade-LAMP amplification

The LAMP amplifications for detection of MPXV Congo Basin clade (**A**) and MPXV West African clade (**B**) were monitored using real-time turbidity, and the corresponding curves of amplicons were displayed in the graphs. Turbidity > 0.1 indicated a positive value. 8 kinetic graphs were obtained at different temperatures (62-69°C, 1°C intervals) with 1×10^4 copies target gene. **A**, The graphs from **e** (66 °C) to **f** (67 °C) showed robust amplification; **B**, the graphs from **d** (65 °C) to **f** (67 °C) showing robust amplification.

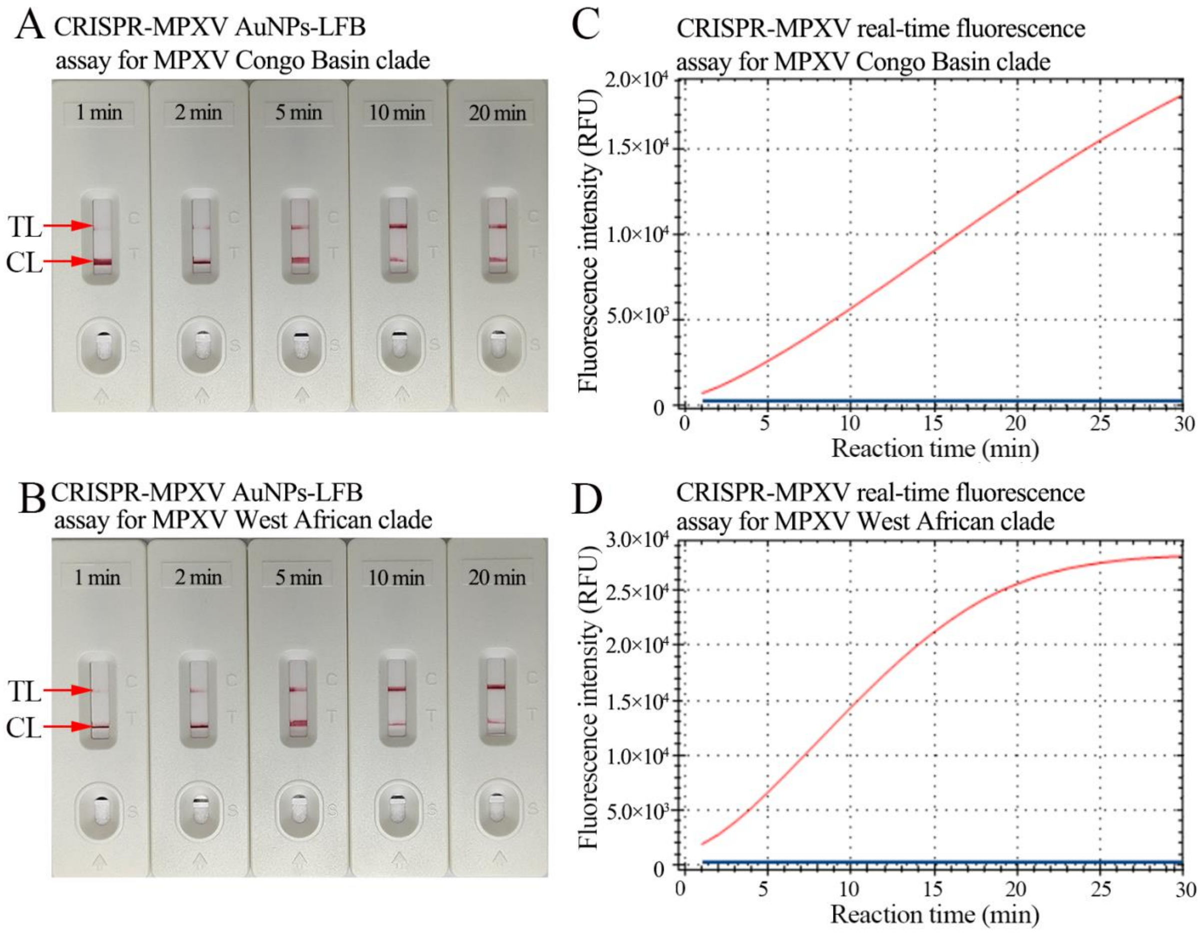

Fig. S4. Optimizing of the reaction time for CRISPR-Cas12b/gRNA cleavage

AuNPs-LFB **(A**, **B)** and real-time fluorescence(**C**, **D**) were used for interpretation of the CRISPR-Cas12b/gRNA cleavage results. LAMP products (2 µl) yielded from 1×10^3 copies of MPXV Congo Basin clade *D14L*-plasmid (**A, C**) or West African clade *ATI*-plasmid (**B, D**) were added to corresponding to CRISPR-Cas12b/gRNA reaction. The remarkable signal at the test line (TL) (**A**, **B**) was appeared within 5 min, and the fluorescent signal was also detected within 5 min. these results indicating that the ssDNA probes were sufficiently cleaved within 5 min.

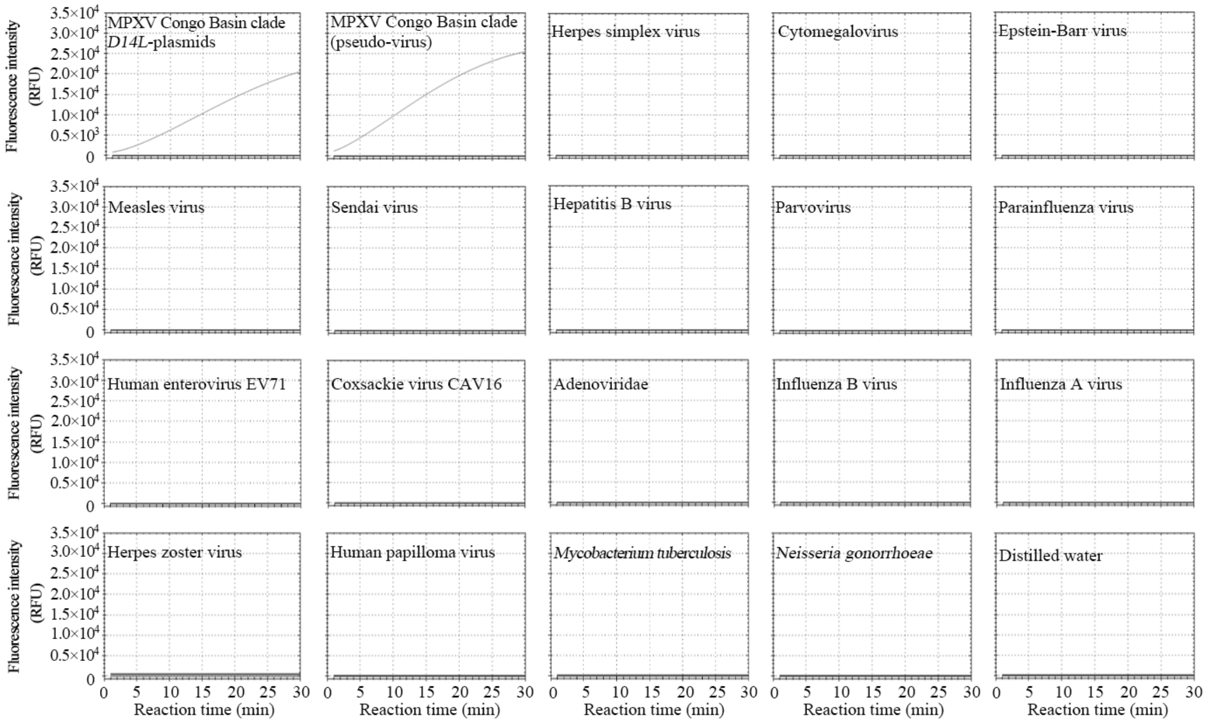

Fig. S5 The specificity of CRISPR-MPXV real-time fluorescence assay for MPXV Congo Basin clade

The LAMP amplification and CRISPR-Cas12b-based real-time fluorescence detection as described above. The MPXV Congo Basin clade-*D14L*-plasmid and MPXV Congo Basin clade pseudo-virus have been used as positive control. No signal was detected in other pathogens.

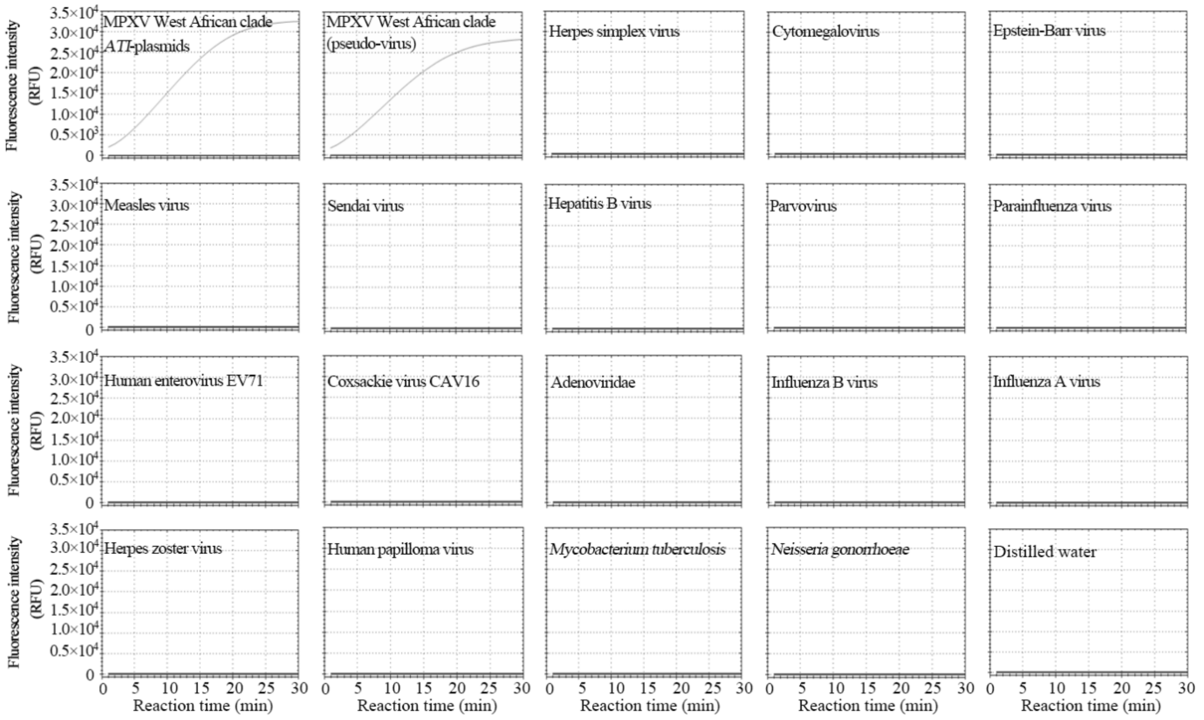

Fig. S6 The specificity of CRISPR-MPXV real-time fluorescence assay for MPXV West African clade

The LAMP amplification and CRISPR-Cas12b-based real-time fluorescence detection as described above. The MPXV West African clade-*ATI*-plasmid and MPXV West African clade pseudo-virus have been used as positive control. No signal was detected in other pathogen.
